# Clinical research without consent in adults in the emergency setting: a review of patient and public views

**DOI:** 10.1186/1472-6939-9-9

**Published:** 2008-04-29

**Authors:** Jan Lecouturier, Helen Rodgers, Gary A Ford, Tim Rapley, Lynne Stobbart, Stephen J Louw, Madeleine J Murtagh

**Affiliations:** 1 Institute of Health and Society, Newcastle University, The Medical School, Framlington Place, Newcastle upon Tyne, UK; 2 Stroke Research Group, Institute for Ageing and Health, Newcastle University, Newcastle upon Tyne, UK; 3 Department of General Internal Medicine, Freeman Hospital, Newcastle upon Tyne, UK

## Abstract

**Background:**

In emergency research, obtaining informed consent can be problematic. Research to develop and improve treatments for patients admitted to hospital with life-threatening and debilitating conditions is much needed yet the issue of research without consent (RWC) raises concerns about unethical practices and the loss of individual autonomy. Consistent with the policy and practice turn towards greater patient and public involvement in health care decisions, in the US, Canada and EU, guidelines and legislation implemented to protect patients and facilitate acute research with adults who are unable to give consent have been developed with little involvement of the lay public. This paper reviews research examining public opinion regarding RWC for research in emergency situations, and whether the rules and regulations permitting research of this kind are in accordance with the views of those who ultimately may be the most affected.

**Methods:**

Seven electronic databases were searched: Medline, Embase, CINAHL, Cochrane Database of Systematic Reviews, Philosopher's Index, Age Info, PsychInfo, Sociological Abstracts and Web of Science. Only those articles pertaining to the views of the public in the US, Canada and EU member states were included. Opinion pieces and those not published in English were excluded.

**Results:**

Considering the wealth of literature on the perspectives of professionals, there was relatively little information about public attitudes. Twelve studies employing a range of research methods were identified. In five of the six questionnaire surveys around half the sample did *not *agree generally with RWC, though paradoxically, a higher percentage would *personally *take part in such a study. Unfortunately most of the studies were not designed to investigate individuals' views in any depth. There also appears to be a level of mistrust of medical research and some patients were more likely to accept an experimental treatment 'outside' of a research protocol.

**Conclusion:**

There are too few data to evaluate whether the rules and regulations permitting RWC protects – or is acceptable to – the public. However, any attempts to engage the public should take place in the context of findings from further basic research to attend to the apparently paradoxical findings of some of the current surveys.

## Background

Few people would disagree with the need to conduct acute clinical research to develop and improve methods of treating patients admitted to hospital with life-threatening and debilitating conditions. In these circumstances obtaining informed consent can be problematic. Patients may initially be unconscious, in shock or have some cognitive impairment, and if the study intervention has a short therapeutic window there is little time to locate a proxy for consent. It follows, therefore, that for research of this kind to proceed with patients who have impaired decision-making capacity, in some patients it must do so without their consent. This illustrates the key ethical dilemma of allowing acute research in which society as a whole will benefit, whilst maintaining respect for the individuals who contribute to that research[1]. Given the turn towards greater patient and public involvement in health care decisions [2] following the events of the 20^th ^century relating to human subject research, research without consent clearly raises concerns about unethical practices [3-5] and the loss of individual autonomy[6] that is "respect for the patient's capacity of self-determination, and exercise of personal choice"[7]. The subject of considerable debate among researchers, medical ethicists and philosophers for a number of years, the overriding objection to waiving individual informed consent is that it erodes patient autonomy. However, some point out autonomy is not the sole applicable ethical principle and those involved in research have a duty – in respect to the principle of justice – to develop potentially beneficial therapies that are available to all populations[8,9]. Others have questioned the equity of excluding patients where individual or proxy consent is not possible and denying them the right to participate and to any potential benefits[10,11]. Opinion regarding informed consent ranges from the view that (in certain circumstances) the process is 'needlessly cruel'[12] and violates the principle of beneficence for some patients[13], to concerns that it should *always *be obtained[14,15] and permitting waiver of consent is a slippery slope toward unethical practices[16]. At the same time, emergency researchers are frustrated by a somewhat paradoxical situation where physicians are free to prescribe, without ethical approval, a medication for their own patients that has not been tested scientifically with that particular medical condition [17-20].

Research without consent (RWC) is currently permissible in certain situations under specific conditions in the US, EU member states, Canada, and Australasia. In the US it has been argued that these regulations (Food and Drug Administration. 21 CFR 50.24 – Exception from Informed Consent) have led to a reduction in the number of emergency medicine studies implemented [21]. Although a published review of journal articles (up to 2004) and FDA information (up to 2003) identified only five studies as being conducted under the US regulations since their implementation in 1996 [22], at a Food and Drug Administration hearing held in October 2006 it was reported that 21 studies had been conducted, were underway or about to commence[23].

In Europe, RWC in emergency situations was permissible in many countries but in 2004 the European Union Directive [24] on clinical research became law across the (then) 25 EU member states. The Directive which was introduced to 'simplify and harmonise the regulation of clinical trials across the EU' [25] makes no provision for RWC in emergency situations. Although at variance with the Directive, Austria, Belgium, France, The Netherlands, Germany and Spain retained their existing national legislation for emergency research: Denmark, Italy, Ireland, Portugal and the UK attempted to adhere to the rules of the Directive [26]. At the end of 2006 the UK issued an amendment to the Clinical Trials Directive [27] to permit RWC in emergency situations with ethical committee approval.

Legislation has been implemented to protect patients and facilitate acute research with adults who are unable to give consent. Public involvement in health, social policy and practice is already well established in the UK [28] and researchers are increasingly expected to involve the public in all stages of the research process. Yet to date the views of patients and the public have not been considered when developing research legislation[29]. This paper reviews research examining public opinion regarding RWC in emergency situations, and whether the rules and regulations permitting research of this kind are in accordance with the views of those who ultimately may be the most affected.

## Methods

Seven electronic databases – Medline, Embase, CINAHL, Cochrane Database of Systematic Reviews, Philosopher's Index, Age Info, PsychInfo, Sociological Abstracts and Web of Science – were searched up to the end of May 2007. The search terms are listed in Table 1. Searches were conducted by JL and articles were individually reviewed by both JL and MM. Any queries over the suitability of studies for inclusion were resolved by a third person (HR).

Only those articles pertaining to the views of the public in the US, Canada and EU member states regarding research in an emergency setting involving adults were included. Opinion pieces and those not published in English were excluded. We recognise the important issues relating specifically to obtaining consent from parents to enrol their children in paediatric research but feel they are beyond the scope of this review.

**Table 1:** Search terms used

		patient* views OR
emergency research OR		patient* perception* OR
emergency medicine OR		patient* opinion* OR
exception from informed consent OR	**AND**	patient* attitude* OR
waiver of informed consent OR		public* views OR
emergency exception OR		public* perception* OR
resuscitation research		public* opinion* OR
		public* attitude*

## Results

Using the terms relating to emergency research and patient/public opinion (Table 1) we elicited 7298 and 13224 articles respectively in the Medline search. Combining these terms resulted in a total of 28 papers; from the titles 24 were deemed to be unsuitable for the review. The search was exploded to ensure greater coverage and a further seven papers were identified using the terms 'emergency research', 'emergency exception' and 'exception from informed consent'. Two further relevant papers were identified from searching the references lists of key articles. Searches in the other databases listed did not elicit any new papers suitable for this review.

### Public attitudes

A search of the published research literature identified six surveys specifically exploring public attitudes to clinical research on human subjects without consent (Tables 2 and 3). One was conducted in the UK[30], the rest in the US[29,31-34]. Two studies canvassed the views of emergency department (ED) patients solely[29,33]; two of ED patients and visitors [31,34]; one of patients from the ED and a geriatric clinic[32]; and one of attendees at an out-patient department[30]. Four of the six sought opinions regarding RWC generally: all asked if the respondent personally would be happy to participate in a study where consent was not obtained. Hypothetical scenarios or descriptions of medical situations where obtaining consent would be difficult were used. Some scenarios introduced the issue of minimal and more than minimal risk.

**Table 2:** Studies reporting public opinion of research without consent – Aspects of study design

**Author**	**Country**	**Study design**	**Sample**	**No of subjects**	**Scenarios/hypothetical studies**	**Measurements**
Smithline & Gerstle (1998) [29]	US	Interview survey	Emergency Dept pts	204	Patient had serious illness	Agree/disagree
					1. Study intervention with minimal absolute risk	
					2. Study intervention with greater than minimal absolute risk	
McClure et al (2003) [31]	US	Interview survey	Emergency Dept pts & visitors	530	1. Study intervention with minimal absolute risk	5-point Likert scale
					2. Study intervention with greater than minimal absolute risk	
					Two study scenarios:	
					1. New therapies for serious bleeding	
					2. Efficacy of public access to defibrillation	
Abboud et al (2006) [32]	US	Interview survey	Emergency Dept pts	207	Patient in cardiac arrest	
			Geriatric Clinic pts	213	Two surgical procedures:	
					1. Less invasive, intravenous line (minimal risk)	4-point Likert scale
					2. More invasive, thoracotomy (>minimal risk)	
					New experimental medicine in three study designs:	
					1. Outside of a study protocol;	
					2. Part of a study protocol;	
					3. In a randomised controlled trial	
Goldstein et al (2007). [33]	US	Interview survey	Emergency Dept pts	473	Patient suffered cardiac arrest or stroke	Not stated
Triner et al (2007) [34]	US	Interview survey	Emergency Dept pts & visitors	497	General description of waiver of and exception from informed consent studies	5-point Likert scale
Booth et al (2005) [30]	UK	Self-completion questionnaire	Out-patients	362	Patient suffered heart attack, stroke or head injury	Yes/No/Don't know
					1. Study intervention with minimal risk	
					2. Study intervention with moderate risk	

**Table 3:** Studies reporting public opinion of research without consent – Findings

**Author**	**Sample**	**No of subjects**	**% agreed with RWC**	**% would personally be enrolled without consent**			**Factors measured**	
						
				**risk not specified**	**minimal risk research**	**> minimal risk research**	**associated with attitude to RWC**	**not associated**
Smithline & Gerstle (1998) [29]	Emergency Dept pts	204	-	-	73	50	Educational status and certain aspects of health status	Age, race, gender, perception of current acute illness.
McClure et al (2003) [31]	Emergency Dept pts & visitors	500+	34	70	75	50	Race	Gender, religion, education, insurance status, knowledge of resuscitation medicine.
Abboud et al (2006) [32]	Emergency Dept pts	207	-	70	88	77	Study design, invasiveness of intervention, patient group	Age, race; marital status, living situation, religion, church attendance, education, having an advance directive
	Geriatric Clinic pts	213		48	63	48	Geriatric clinic patients only – gender and health status	
Goldstein et al (2007). [33]	Emergency Dept pts	473	51	57	-	-		Age, gender, race, ethnicity, education, insurance status, religion, confidence in current therapies & knowledge of requirements for RWC studies
Triner et al (2007) [34]	Emergency Dept pts & visitors	497	42	50	-	-	Age, Gender, ethnicity	Marital status, education.
Booth et al (2005) ^12^	Out-patients	362	84	-	92	67	None reported	None reported

not measured

In the two studies of ED patients and visitors[31,34], around half supported RWC but the proportions happy to take part in such a study differed (70% v 50%). Participants in the UK study responded more positively to RWC, but favoured a 'deferred' approach, suggesting consent should be obtained from a relative (82%) or the patient (90%) as soon as possible following recruitment to the trial[30]. The authors point out their sample of out-patients may respond more positively to the concept than those who are not regularly attending hospital. The majority of ED patients stated they would participate without consent but the proportions ranged from 57%[33] (risk not specified) to 73%[29] and 88%[32] (minimal risk). For patients attending a geriatric clinic, support for RWC decreased from 67% to 48% and for ED patients 83% to 70% when the experimental drug was being tested as part of a randomised controlled trial rather than 'outside' of a study protocol[32]. As participants had a high level of trust in their doctor to act in the patients' best interest (91–97%), the authors state these findings may reflect distrust of the research process. The association between degree of risk and agreement with RWC demonstrated in three of the studies[29,32,30] is supported by a focus group study where the levels of risk described in the study scenarios were shown to have a bearing on public attitudes, as was benefit and personal experience of research or health professionals (directly or through a family member)[35].

Other studies were identified where – although not the main focus – data relating to RWC was collected. Two UK studies were identified involving consumers in the design of an RCT: participants supported a waiver of consent procedure for incapacitated patients for a hypothetical thrombolysis trial but only in the absence of a relative willing to act as a proxy[36] whilst for a lower risk trial to evaluate routine oxygen supplementation there was greater agreement with waiver of consent because of a recognition of the stress of decision making upon relatives at a difficult time[37].

Another source of information is the data collected from community consultation, a regulatory requirement of exception from informed consent in the US. To canvass public views, one recently published study sampled three sets of community representatives (1136) and found that over half (58%) agreed that waiver of consent was justified in that particular project which the authors describe as low risk[38]. A qualitative study conducted to ascertain the preferences of (12) stroke patients' and their family members about consent in emergency research reported all but one were happy for the doctor to make the decision about enrolment to the trial in the absence of a surrogate to provide informed consent[39]. Analysis of community consultation data revealed one public concern was that there should be an opt-out mechanism to allow prospective refusal or the identification of uninterested patients[40].

Despite the fact that clinical research involving humans has been permitted without consent for many years – over 10 years in the US using the Exception and Waiver regulations – we were unable to identify any papers reporting the views of patients (or of their relatives) involved in any of these studies. Data has been collected regarding the views of families whose relatives were involved in studies using deferred consent. This approach (sometimes referred to as 'retrospective consent') depends upon obtaining consent from the patient or legal representative after the patient has been entered into the trial[41]. The consent – when it is obtained – is actually consent to remain in the study. In one study, out of 531 patients enrolled using this approach, only six families reacted negatively[42]. In 1993 deferred consent was deemed no longer acceptable under USA regulations[43] because it was not legally equivalent to informed consent[44].

There are published studies exploring the perspectives of those approached in an acute situation for research participation with regard to informed consent. One study of 181 patients with acute myocardial infarction reported less than half (41%) found it acceptable to have to decide whether or not to enrol in those circumstances[45]. In the open comments, those who did not find it acceptable gave the following reasons: 'it was impossible to make a decision under the circumstances; that they did not want to be asked to make any decisions under the circumstances; or that they did not approve of the randomisation'. Of those who thought it unacceptable only two felt the decision should rest with a physician. In a qualitative study of 31 patients' experiences of the consent procedure in an acute myocardial infarction trial the majority would have been willing for a physician to make the decision had they been too ill to approach for consent [46].

### Protection of subjects in the absence of informed consent

#### Community consultation

One requirement of the Waiver of, and Exception from, Informed Consent regulations in the US is community consultation, a two-way communication between the community and research team. Proposed as an additional protection for patients, its purpose is to elicit the opinions and obtain input from the community where the research will be conducted, about the proposed study and the exception from informed consent. This information is fed back to the Institutional Review Board for consideration before the study is approved [40]. Despite the fact there is no clearly defined or published regulatory intent, those working in the field of emergency medicine have proffered their own interpretations of community consultation. It has been considered to be a means of promoting trust within the community [8]. In the absence of the one to one encounter between participant and researcher under 'normal' conditions, the latter can disclose details of the project to members of the community, document and respond to any concerns [47]. An assumed benefit is that this will deter researchers from planning to conduct research of which they fear the public may disapprove[48]. This exercise has generated a significant amount of published literature, especially in relation to problems with its implementation [35,38,47,49-52]

As researchers are not required to demonstrate how successful this exercise has been, little has been published about its efficacy or its acceptability to the general public. One focus group study asked participants to consider how researchers should proceed with research when the potential subject cannot give consent, has no family member and there is no time to contact anyone[35]. The authors reported that the participants struggled with this conceptually and 'the facilitator had to repeatedly refocus the participants on this issue.'. This raises the question that if members of the community have this problem in focus groups how will others fare in large public meetings where they may have less time and less opportunity to gain a full understanding of research without consent and the situations that demand it. A survey reported that 5% of the 530 ED respondents to the survey were aware of current research in their community using the exception from informed consent[31]. Respondents were also asked if they regarded community consultation and public notification as a reasonable substitute for patient consent, although within the FDA regulations this is not the intention. However, 45% agreed that these exercises were a reasonable substitute for patient consent and 49% would attend a community meeting. As the authors state, in the light of these findings community consultations may not be the most effective method of meeting the ethical requirements of the regulations. In the UK – where community consultation is not a requirement for emergency research – 62% of patients surveyed felt public meetings were a practical way forward and 35% stated they would attend[30].

## Discussion

The available data from the US reveals around half of those surveyed do *not *agree generally with research without consent in emergency situations [31,34,33,35]. Paradoxically, however, a higher percentage would *personally *take part in such a study. Unfortunately the studies were not designed to investigate in any depth individuals' views regarding participation in studies where consent was waived. The association between level of risk, or invasiveness of study intervention, and support for research without consent is understandable, but that with study design is not. Patients were more likely to accept an experimental treatment 'outside' of a research protocol without giving consent than in a randomised controlled trial, but again it is not known why this is so. In reality, patients who participate in an organised research study that has undergone scientific and ethical review are more protected than those whose doctor prescribes a treatment unproven with their condition[32]. However, it is not clear whether the public are aware of the protections in place. One explanation is that the use of randomisation may be problematic for the lay person. Concern about randomisation and a preference for the doctor to choose treatment have been most commonly cited as reasons for refusing to enrol in one study[53]. It is not clear whether this stemmed from a lack of, or a full understanding of randomisation. In another trial the authors felt that some of their study respondents fully understood but disliked and resisted concepts such as placebo and randomisation, for example[54]. They believe this may be because the idea that the physician does not know – or knows and does not randomise them to – the 'best' treatment, sits uncomfortably with some people and conflicts with their views of, and trust in, medical care.

Considering the wealth of literature on the perspectives of professionals, ranging from bio-ethicists to physician investigators, there is relatively little information about public attitudes toward research without consent or of their views on how to engender the development of new treatments whilst protecting vulnerable participants. Reasons for a lack of support for research without consent may be due to a general lack of awareness of how few treatment options available there are for some conditions and that many current therapies are poorly supported by evidence or have poor outcomes[23], although research in this area is scarce. This is an interesting point, however, considering the backlash against protectionism with some AIDS and cancer sufferers demanding the right to participate in clinical trials of new medications[55].

Additionally, though there are very few data from other countries with which to draw comparisons, the American public may be more suspicious and less supportive of medical research. A US focus group study published in 2005 reported that participants were aware of incidences of unethical research but felt these were now "less common" and that "research ethics had improved"[35]. Participants in the same study also believed that medical staff often used any surplus blood for research without informing the patient or seeking their permission. A focus group study of ED patients and visitors reported that almost half (49%) equated research participants to 'human guinea pigs'[56].

There are too few data to evaluate whether the US community consultation requirement protects – or is acceptable to – study participants and there is a need for research in this area. The only concern in relation to research without consent identified from communications with the public was a system to opt out. There is some public support for patients' wishes regarding research participation to be recorded on cards or wristbands[32].

Professionals believe that too much emphasis is placed on informed consent, which does not in itself guarantee ethical research[57-59]. Those advocating a move from a reliance on informed consent as a protection for research subjects suggest improved study design to ensure ethical clinical research[60]. The ethics of clinical research should be judged on: the value of the research; scientific validity; fair subject selection; favourable risk-benefit ratio; independent review; informed consent; and respect for participants [58,61]. Although still part of the ethical framework, when circumstances preclude obtaining informed consent there should be clinical equipoise which will ensure 'the subject is not worse off by enrolling'[58]. Clearly these should be standard requirements of every research study but they may not go far enough to satisfy those opposed to, or uncomfortable with, research without consent.

For research without consent to be accepted as a necessity in certain circumstances we suggest there is a need for public involvement in the debate. Any programmes of involvement need to attend to the apparently paradoxical findings of some of the current surveys. For example, if people are more likely to accept experimental treatments outside of a research environment than within one, we clearly need to increase public trust in medical research. This may be bolstered by providing information about the rationale for certain study designs, and highlighting the ethical assessments that clinical trials currently undertake to be approved. We may also need to increase awareness of the standard and efficacy of current therapies and of the necessity to evaluate and develop new ones within the framework of randomised controlled trials. Additionally, a system that enables people to opt out of clinical research might be another measure to reassure the public and protect patient autonomy but this warrants further investigation. However, any attempts to engage the public in such a programme of the 'public understanding of research', should take place in the context of findings from further basic research. We need to understand the factors that promote and inhibit trust in experimental treatments and the potential concerns that patients and the public have around such treatments in randomized controlled trials.

The findings from the survey research raised other interesting issues. Firstly a survey in the UK found that two-thirds of people felt that community consultation could be a practical solution but significantly less of them were willing to attend such meetings. So actually undertaking a community consultation that produces 'democratic' and 'representative' directives for research without consent appears problematic from the outset. Secondly, a survey in the US found that more people were willing to take part in a study when speaking about 'themselves' – when thinking about whether they would personally take part – than when speaking in general terms. When people think about experimental treatments in relation to a potential future moment in their own illness biography they are a lot more accepting of research without consent. We need to be aware of how we frame our research questions and how respondents are making sense of them – are we asking them to offer their views about research without consent in relation to different potential illness episodes involving them or a significant other, or at a more abstract level, about their 'faith' in the idea of research without consent. Additionally, how does this relate to the ideals of experimental research, that people should not be encouraged to take part for personal benefit per se but rather to enable the future benefit of future patients? Finally, no research has focused on the experiences and views of patients who have actually taken part in research without consent. Rather than solely relying on people engaging in hypothetical 'thought experiments', it is essential to generate additional evidence from patients enrolled in such studies to help to inform the continuing ethical dilemma of research without consent.

## Conclusion

The question as to whether current rules and regulations permitting research without consent is acceptable to, or protects, the public remains unanswered. We suggest there is a need for accessible and comprehensive public information about medical research and for public involvement in the debate surrounding research without consent. Before this happens, however, we must understand the factors that influence patient and public trust in medical research.

## Competing interests

HR is Deputy Director of the UK Stroke Research Network where she is responsible for patient, carer and public involvement. She is a principal investigator for acute stroke trials. GF is Director of the UK Stroke Research Network. He is a steering committee member and grantholder for commercial and academic acute stroke trials and an advisory board member for acute stroke treatments.

## Authors' contributions

MM, LS, HR, GF and SL designed the main study and obtained funding. JL and MM developed the search strategy for the review. JL conducted the review and MM acted as second reviewer. JL wrote the first draft which was revised by all the other members of the study team. All authors have read and approved the final manuscript.

## Pre-publication history

The pre-publication history for this paper can be accessed here:


